# Longitudinal changes in mobility among nonagenarians: the Vitality 90+ Study

**DOI:** 10.1186/s12877-015-0116-y

**Published:** 2015-10-15

**Authors:** Kristina Tiainen, Jani Raitanen, Elina Vaara, Antti Hervonen, Marja Jylhä

**Affiliations:** School of Health Sciences and Gerontology Research Center, University of Tampere, 33014 Tampere, Finland; UKK-Institute for Health Promotion Research, P.O. Box 30, 33501 Tampere, Finland; Department of Social Research, University of Helsinki, P.O. Box 54, 00014 Helsinki, Finland; Institute for Advanced Social Research, University of Tampere, 33014 Tampere, Finland

**Keywords:** Functional performance, Physical functioning, Functional disability, Longitudinal study, Aging

## Abstract

**Background:**

Several studies have focused on predictors of mobility limitations and disabilities. Yet little is known about the pace and patterns of mobility changes among very old people. This study examined changes in functional mobility among individuals aged 90 years and older during a 2-9-year follow-up. In addition, we were interested in the patterns of mobility changes.

**Methods:**

Data were collected through a mailed questionnaire in the years 2001, 2003, 2007 and 2010. The study population (*n* = 948) consisted of individuals from three cohorts (2001, 2003, 2007) who participated in at least two survey rounds and answered the mobility questions. The length of the follow-up varied from 2–9 years between individuals as well as according to how many times an individual took part in the survey. Multilevel ordinal logistic regression analysis was used to evaluate the effects of time, age, gender, cohort and chronic conditions on changes in mobility.

**Results:**

At the baseline, “younger” old people, men and individuals in the cohorts for 2003 and 2007 had significantly better mobility compared with women, older individuals and individuals in the 2001 cohort. In addition, individuals with fewer chronic conditions had better mobility than those with more diseases. Mobility declined for most of the participants during the follow-up. The difference in the change in mobility over time for gender, age or chronic conditions was not statistically significant. The analyses were performed with a subgroup of participants aged 90–91 years at the baseline, and results did not differ substantially from the results for the entire study sample. However, the effect of chronic conditions on the change in mobility was statistically significant among participants aged 90-91years.

**Conclusions:**

No differences were observed in the rate of mobility decline over time between age or gender. The effect of chronic conditions on the change in mobility was significant only among individuals aged 90–91 years. The prevention efforts are important and should focus even more, also among the oldest-old, on additional modifiable risk factors such as maintaining muscle strength.

## Background

Mobility is defined as a person’s ability to move safely and independently from one place to another [1]. If a person’s resources, such as muscle strength and balance, are at a lower level than the demands of the environment, it results in mobility limitations and disabilities, as well as in difficulties in functional performance. The first signs of functional limitations can often be seen in mobility in complex and more demanding functions such as walking outside and using stairs; later limitations surface when carrying out simpler tasks such as rising from a chair or in dressing and undressing oneself [2]. A decline in mobility predicts limitations and disabilities in the activities of daily living, ADL [3], but also restricts a person’s independence and decreases the quality of life [4, 5]. An extensive amount of research has been carried out with the goal of identifying the predictors of mobility limitations and disabilities [3, 6]. However, little is known about the pace and pattern of mobility changes.

The oldest-old represent the most rapidly growing age group of the population in developed countries and the prevalence of mobility disability among them is high [7]. However, only limited data are available regarding the changes in mobility among nonagenarians. Earlier studies among people younger than 85 years of age have shown that an average of 60 % of mobility disability develops gradually over the years, rather than occurring abruptly [8, 9]. Among people aged 85 years and older the slow, gradual development of disability is even more frequent [9]. Earlier results with respect to the effects of age and gender on changes in mobility are contradictory and often focused on “younger” old people [10, 11]. In the study by Holstein and colleagues [10], half of the surviving participants aged 70 to 95 years had unchanged functional performance during the eight-year follow-up. Although the decline in functional performance was particularly high among persons aged 80 years and older, the changes in functional ability were not related to age or gender among people aged 70 years and older. The most substantial decrease was seen in mobility and in the more outgoing instrumental activities of daily living. It should nevertheless be noted that participants in this study were non-institutionalized. However, in the study by Ahacic and colleagues [11] age was related to the change in mobility among men and women aged 77 years and older; older age was associated with the increased odds for mobility limitations. To understand the pathways and patterns of mobility changes, we need well-designed longitudinal studies of older people, including both non-institutionalized as well as institutionalized individuals. Prevention efforts are important and should be focus on the right thing. Findings from the longitudinal study will offer the background information to design the successful prevention and intervention.

The purpose of this study was to examine the effects of age, gender, cohort and chronic conditions on changes in functional mobility (condition improved, remained unchanged, declined) among individuals aged 90 years and older during the 2-9-year follow-up. In addition, we were interested in patterns of mobility changes among nonagenarians.

## Methods

### Participants

**Fig. 1:**
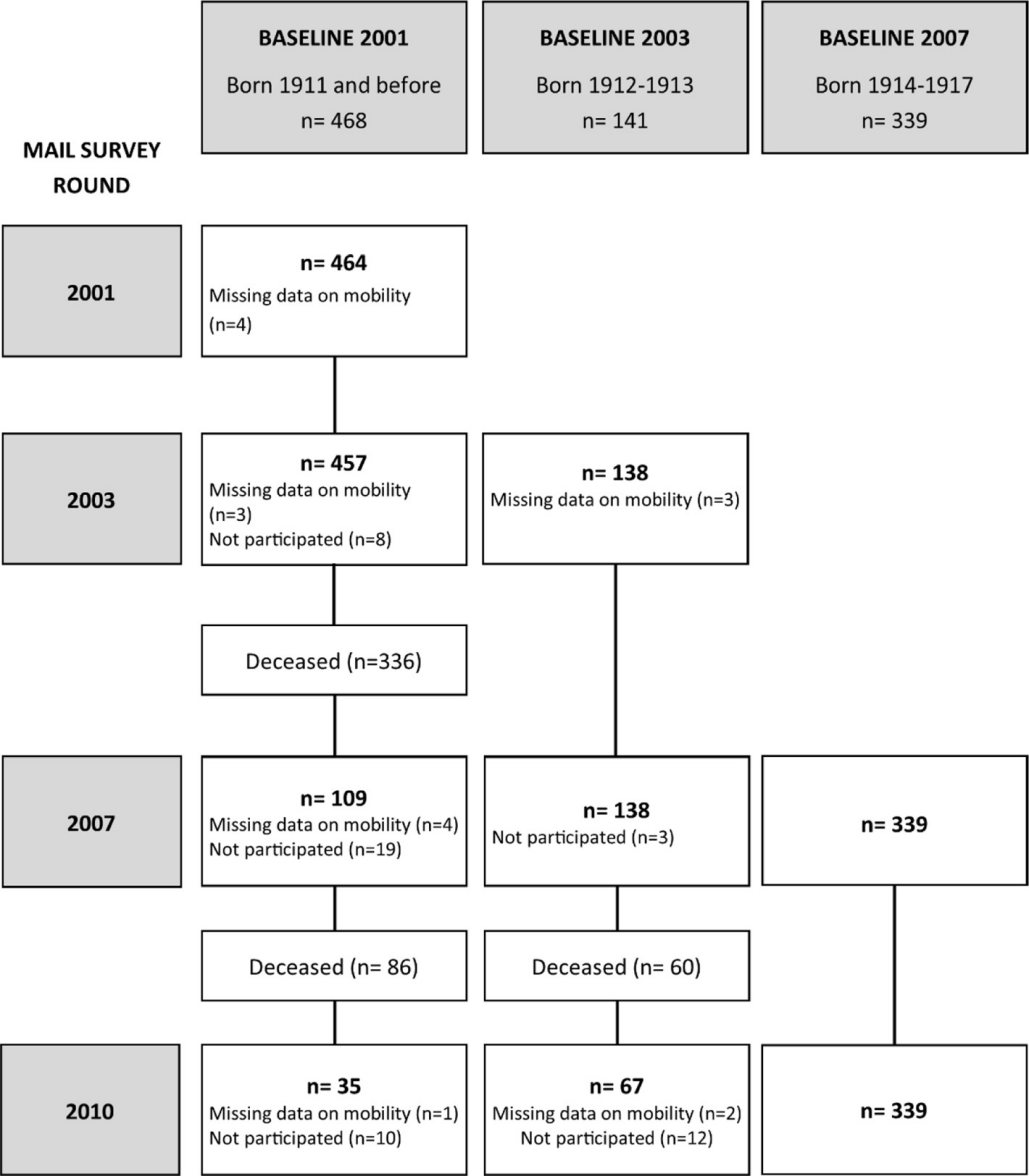
The number of participants and their follow-up times started in 2001, 2003 and 2007 (*n* = 948)

This study is part of the Vitality 90+ Study, which is a population-based research program dealing with nonagenarians in Tampere [12, 13]. In the Vitality 90+ Study, mailed questionnaires concerning wellbeing and functioning were sent out in the years 2001–2010 to all individuals aged 90 years and older, irrespective of health or dwelling place, who, according to the Tampere City Population Register, were living in the Tampere area. In the present study, the sample consists of individuals from three cohorts. The cohort is defined here as the year when the participant answered the questionnaire for the first time, the baseline. At first, all individuals in the 2001 cohort (born in 1911 or before, *n* = 1129, age range 90–104 years) were included in the study. To expand the sample size individuals from the cohort for 2003 (born in 1912–1913, *n* = 1113, age range 90–91 years) and 2007 (born in 1914–1917, *n* = 1146, age range 90–95 years) were also added to the study sample. The final sample of the present study consisted of individuals from the cohorts for 2001, 2003, 2007 who participated in at least two survey rounds and answered the mobility questions. From the 2001 cohort 468 individuals fulfilled these two criteria; the corresponding numbers in the cohorts for 2003 and 2007 were 141 and 339, respectively. The final sample (*n* = 948) consisted of 782 women and 166 men (Table 1, Fig. 1). Both the length of the follow-up (2–9 years) and the number of times the participants took part in the survey varied between individuals. Mortality was the most important cause of attrition (Fig. 1).

**Table 1:** Characteristics of the study population (*n* = 948) in the Vitality 90+ Study

	Cohort 2001			Cohort 2003			Cohort 2007		
	Men (*n* = 81)	Women (*n* = 387)	All (*n* = 468)	Men (*n* = 28)	Women (*n* = 113)	All (*n* = 141)	Men (*n* = 57)	Women (*n* = 282)	All (*n* = 339)
Age, median (IQR), years	91 (90–92)	91 (90–93)	91 (90–93)	90 (90–91)	90 (90–91)	90 (90–91)	91 (90–92)	91 (90–92)	91 (90–92)
Chronic conditions, median (IQR), number	1 (0–2)	1 (1–1)	1 (1–2)	1 (0–2)	1 (0.5–2)	1 (0–2)	1 (1–2)	2 (1–2)	1 (1–2)
Mobility score at baseline, median (IQR)	11 (8–12)	9 (6–11)	9 (6–11)	12 (11–12)	11 (8–12)	11 (9–12)	11 (10–12)	10 (7–11)	10 (7–12)
Respondent (%)									
Participant	91	79	81	96	92	93	97	88	89
Proxy	6	21	18	4	8	7	3	12	10
Unknown	3	<1	1	-	-	-	-	-	<1
Residence (%)									
Community	91	71	75	89	82	84	91	76	78
Institution	8	29	25	11	18	16	9	24	22
Unknown	1	-	<1	-	-	-	-	<1	<1

*IQR* interquartile ranges

In each survey round, the participation rate was high, and varied from 79 % to 86 % depending on the year. The response rate in the mobility questions was also high, varying from 96 % to 99 %. Persons unable to fill out the questionnaire were instructed to ask for help from a family member, a caregiver, or a friend. If a person was unable to select an answer, a caregiver or a family member was asked to participate as a proxy. During the years of the study, the proportion of total proxy participants varied from 15 % to 23 % for the total population.

The study design was approved by the Ethics Committee of the Pirkanmaa Hospital District and the Ethics Committee of the Tampere Health Center. All participants gave their written informed consent.

### Variables

In each of the four mailed-survey rounds (2001, 2003, 2007, and 2010), participants were asked whether they were able to perform four mobility activities: getting in and out of bed, moving about indoors, walking 400 meters, and using stairs. The alternative answers were, (1) yes, without difficulty; (2) yes, with difficulty; (3) only with help; and (4) not at all. For the mobility score these were coded as: 3 = yes, without difficulty, 2 = yes, with difficulty, 1 = only with help, 0 = not at all. The mobility score varied from 0, unable to perform any of the tasks, to 12, can do all four tasks without difficulty. This score was used in the subsequent statistical analysis to describe the level of mobility.

Gender, cohort, baseline age and number of chronic conditions (0–7) were studied as potential factors that could have an effect on mobility over time. The participants were asked whether a doctor had told them that they had had any of the following seven chronic conditions: heart disease, cancer (except basal cell carcinoma), diabetes, dementia, stroke, depression or hip fracture. The number of chronic conditions (0–7) at the baseline was used in the statistical analysis.

### Statistical analysis

The characteristics of the study population were described in terms of medians and interquartile ranges (IQR).The mean decline in the mobility score was the difference between two consecutive points in time (mobility score at baseline and follow-up). Multilevel ordinal logistic regression analysis was used to evaluate the effect of time, age, gender, cohort and chronic conditions and their interactions with time on the changes in mobility during the follow-up. The main effects in the models showed the differences between groups at the baseline. The difference in the change of mobility over time was tested using an interaction term between an independent variable (age, gender, cohort, chronic conditions at baseline) and time. Random intercept and random coefficient models were performed and, based on the goodness-of-fit, the random coefficient model was chosen. The linear regression model could not be used because the assumption of normally distributed residuals was not fulfilled.

We performed four different models. In Model 1, the independent variables were age, time and their interaction. In Model 2, gender and its interaction with time was added and in Model 3 the cohort and its interaction with time was included in the model. In Model 4, all of the above-mentioned variables and the sum of chronic conditions at the baseline and their interaction with time were analysed (*n* = 934, 14 participants did not have information about chronic conditions). All the analyses were performed for two groups, first for all participants, *n* = 948, and then for the subgroup of all individuals aged 90–91 years at the baseline, *n* = 613 (cohort 2001 *n* = 265, cohort 2003 *n* = 141, cohort 2007 *n* = 209). Two individuals were excluded from the analysis due to missing data on chronic conditions.

Ordinal logistic regression is an extension of binary logistic regression and the results can be expressed as proportional odds ratios (POR) and 95 % confidence intervals (CI). Each POR may be interpreted as the effect of the variable on the odds of being in a higher category of the outcome (the higher the better) across the entire range of values it takes. POR and 95 % CI were used to assess the impact of the factors related to the level of mobility at the baseline and on the change of mobility during the follow-up. In the analysis, age, time, number of chronic conditions and their interactions with time were standardized (mean = 0, standard deviation = 1) so that the relative effects could be directly compared. The dichotomy variables (gender, cohort) were not standardized. The goodness-of-fit of the models (1–4) to the data was evaluated with the log likelihood ratio test. The level of statistical significance was set at *p* < 0.05. All of the analyses were performed with Stata software, version 13.0 for Windows, using the generalized linear latent and mixed model (gllamm) framework for multilevel analysis [14].

## Results

**Fig. 2:**
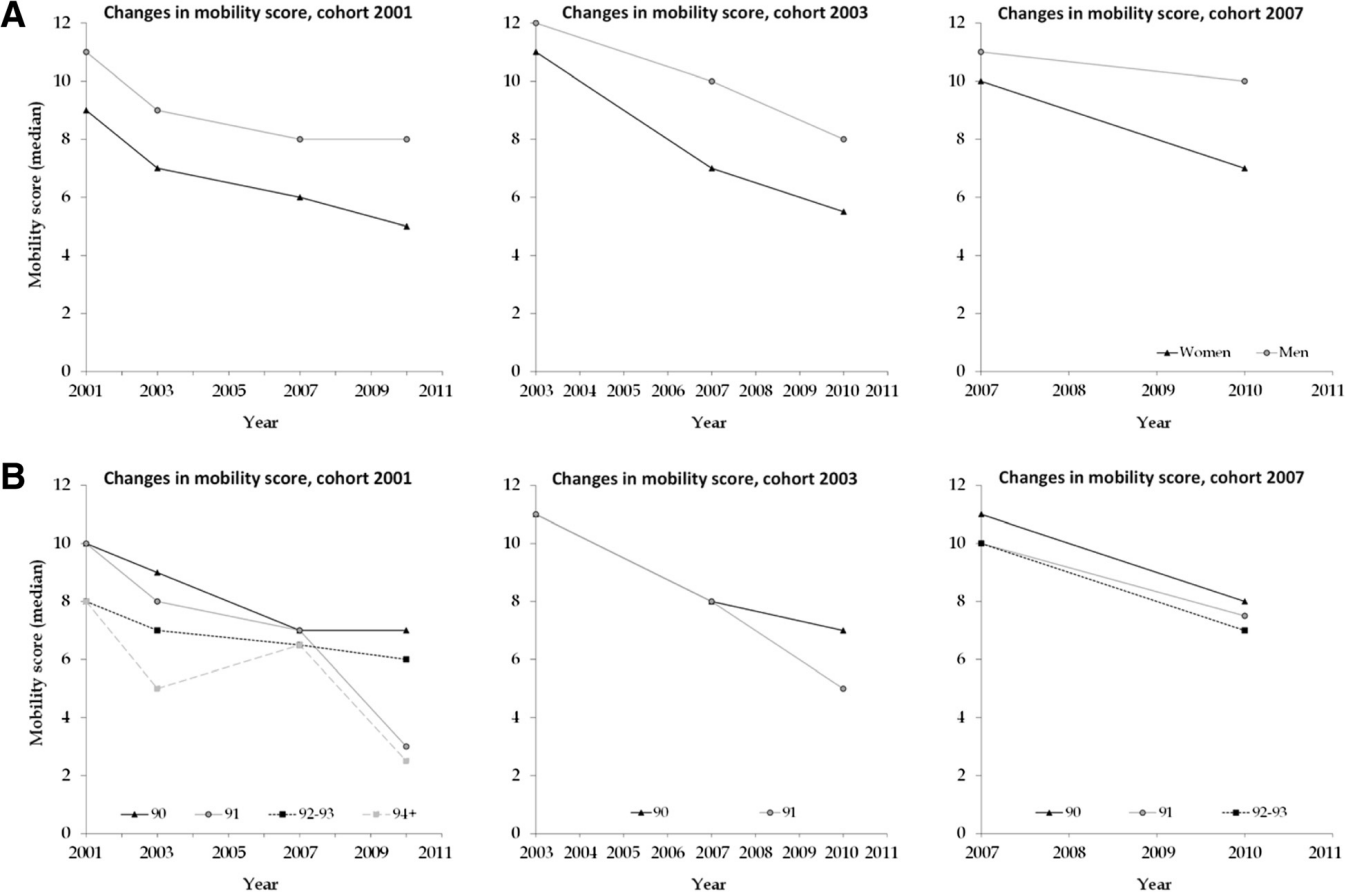
Changes in mobility score during the follow-up: **a** among men and women in different cohorts, **b** in the different cohorts and age groups

The median age at the baseline was 90 or 91 years depending on the cohort (Table 1). In the 2001 cohort age at the baseline varied from 90 to 104, in the 2003 cohort from 90 to 91 and in the 2007 cohort from 90 to 93. The median mobility score (IQR) at the baseline for the participants in the cohorts for 2001, 2003 and 2007 was 9 (6–11), 11 (9–12) and 10 (7–12), respectively (Table 1, Fig. 2a). At the baseline, over 50 % of the participants had a mobility score of 10–12 and fewer than 10 % had a very low mobility score of 0–2. According to Model 4 (Table 2), “younger” old people (POR = 0.60, *p* < 0.001), men (POR = 8.21, *p* < 0.001), individuals with fewer chronic conditions (POR = 0.36, *p* < 0.001) as well as individuals in the 2003 (POR = 4.51, *p* < 0.001) and the 2007 cohorts (POR = 1.99, *p* = 0.009) had significantly better mobility compared with women, older individuals, those who had more chronic conditions and individuals in the 2001 cohort, at the baseline situation.

**Table 2:** The effect of age, time, gender, cohort and chronic conditions on mobility during the follow-up

	Model 1 (*n* = 948)		Model 2 (*n* = 948)		Model 3 (*n* = 948)		Model 4 (*n* = 934)	
	POR (95 % CI)	*p*-value	POR (95 % CI)	*p*-value	POR (95 % CI)	*p*-value	POR (95 % CI)	*p*-value
Age_baseline_	0.47 (0.37–0.76)	<0.001	0.51 (0.40–0.65)	<0.001	0.59 (0.46–0.75)	<0.001	0.60 (0.47–0.77)	<0.001
Time	1.50 (0.00–1120)	0.91	2.24 (0.00–1752)	0.81	11.4 (0.01–11369)	0.49	4.66 (0.00–5733)	0.67
Age_baseline_ * Time	0.14 (0.00–108)	0.57	0.10 (0.00–78.4)	0.50	0.02 (0.00–24.0)	0.29	0.06 (0.00–64.7)	0.42
Gender (ref = women)			9.10 (4.72–17.5)	<0.001	9.64 (4.72–17.5)	<0.001	8.21 (4.32–15.6)	<0.001
Gender * Time			0.88 (0.74–1.05)	0.17	0.87 (0.73–1.03)	0.10	0.87 (0.73–1.03)	0.11
BL2003 (ref = BL2001)					5.02 (2.33–10.8)	<0.001	4.51 (2.16–9.44)	<0.001
BL2007 (ref = BL2001)					1.73 (1.01–2.95)	0.044	1.99 (1.18–3.33)	0.009
BL2003 * Time					0.77 (0.62–0.96)	0.021	0.79 (0.63–0.98)	0.032
BL2007 * Time					0.74 (0.65–0.83)	<0.001	0.74 (0.65–0.84)	<0.001
Chronic conditions							0.36 (0.29–0.46)	<0.001
Chronic conditions * Time							1.13 (0.92–1.38)	0.24

*POR* proportional odds ratios, *95 % CI* 95 % confidence intervals, *BL* base line, year when the participant answered the questionnaire for the first time

Model 1: age, time and their interaction

Model 2: age, time, gender and their interaction with time

Model 3: age, time, gender, cohort and their interaction with time

Model 4: age, time, gender, cohort, number of chronic conditions (0–7) and their interaction with time (*n* = 934, 14 participants did not have information about chronic conditions)

During the follow-up, mobility declined for most of the participants (Fig. 2). The mean decline in the level of mobility per year varied between 0.52-0.92 depending on the cohort and the follow-up time. The decline was more common among women than among men, especially when the follow-up time increased. However, at the same time, there were also individuals whose mobility improved during the follow-up, although they were a minority, and the improvements in mobility were more common among women than among men. The difference in the mobility decline over time for gender or age was not significant (Table 2). Also, Fig. 2a and b show that the decline in mobility did not differ between gender and age. The participants in the cohorts for 2003 and 2007 had a significantly faster decline in mobility than individuals in the 2001 cohort (2003 vs. 2001 POR = 0.79, *p* = 0.032 and 2007 vs. 2001, POR = 0.74, *p* < 0.001, Table 2, Model 4). The change in mobility was not dependent on the number of chronic conditions (Table 2). No essential differences in significances were observed when comparing Models 1–4 (Table 2). Model 4 was chosen because the log likelihood ratio test observed that Model 4 fit statistically significantly (*p* < 0.001) better to the sample when compared to Model 3.

We also conducted the analyses separately in the subsample of men and women aged 90–91 years at the baseline (*n* = 613). The median mobility score (IQR) at the baseline for the 2001 cohort was 10 (7–12), for the 2003 cohort 11 (9–12) and 10 (8–12) for individuals in the 2007 cohort (Fig. 2a, b). At the baseline, men (POR = 10.4, *p* < 0.001) and persons who had fewer chronic conditions (POR = 0.32, *p* < 0.001) had better mobility compared with women and those who had more chronic conditions (Table 3, Model 4). The significant difference between cohorts can also be seen in a subsample; individuals in the 2003 cohort (POR = 4.04, *p* < 0.001) had significantly better mobility than individuals in the 2001 cohort.

**Table 3:** The effect of age, time, gender, cohort and chronic conditions on mobility during the follow-up among participants aged 90–91 years

	Model 1 (*n* = 613)		Model 2 (*n* = 613)		Model 3 (*n* = 613)		Model 4 (*n* = 604)	
	POR (95 % CI)	*p*-value	POR (95 % CI)	*p*-value	POR (95 % CI)	*p*-value	POR (95 % CI)	*p*-value
Age_baseline_ (ref = 90 yo)	0.75 (0.40–1.40)	0.37	0.81 (0.44–1.49)	0.49	0.78 (0.42–1.44)	0.43	0.81 (0.45–1.45)	0.47
Time	0.23 (0.18–0.30)	<0.001	0.24 (0.19–0.32)	<0.001	0.30 (0.22–0.39)	<0.001	0.24 (0.17-0.34)	<0.001
Age_baseline_ * Time	0.94 (0.78–1.13)	0.53	0.94 (0.78–1.13)	0.52	0.95 (0.79–1.15)	0.60	0.95 (0.79-1.14)	0.59
Gender (ref = women)			11.5 (5.16–25.6)	<0.001	12.5 (5.57–28.3)	<0.001	10.4 (4.82–22.5)	<0.001
Gender * Time			0.88 (0.73–1.07)	0.19	0.86 (0.71–1.04)	0.12	0.87 (0.72–1.05)	0.16
BL2003 (ref = BL2001)					4.90 (2.16–11.1)	<0.001	4.04 (1.86–8.80)	<0.001
BL2007 (ref = BL2001)					1.57 (0.78–3.15)	0.21	1.69 (0.87–3.30)	0.12
BL2003 * Time					0.77 (0.61–0.98)	0.030	0.80 (0.64–1.01)	0.059
BL2007 * Time					0.75 (0.64-0.88)	<0.001	0.75 (0.64–0.88)	<0.001
Chronic conditions							0.32 (0.24–0.44)	<0.001
Chronic conditions * Time							1.29 (1.03–1.62)	0.027

*POR* proportional odds ratios, *95 % CI* 95 % confidence intervals, *BL* base line, year when the participant answered the questionnaire for the first time

Model 1: age, time and their interaction

Model 2: age, time, gender and their interaction with time

Model 3: age, time, gender, cohort and their interaction with time

Model 4: age, time, gender, cohort, number of chronic conditions (0–7) and their interaction with time (*n* = 604, 9 participants did not have information about chronic conditions)

Among 90-91-year-old people the mean decline in the level of mobility per year varied between 0.60-0.84, depending on the follow-up time. The difference in the mobility decline over time for gender or age groups (90 vs 91 years of age) was not significant (Table 3). The estimated odds for the change in mobility for a subject in the group of 90-year-old individuals is multiplied by 0.24 every year and the estimated odds for a subject in the group of those aged 91 years is multiplied by 0.23 every year. In terms of percentage decreases in estimated odds, 100 % (1-POR), the odds decreased 76 %/year in the group aged 90 years and 77 %/year among 91-year-old people (Table 3). Mobility declined significantly faster among individuals in the 2007 cohort (POR = 0.75, *p* < 0.001) and among those who had fewer chronic conditions at the baseline (POR = 1.29, *p* = 0.027) compared with individuals in the 2001 cohort and those having more chronic conditions, respectively (Table 3, Model 4). Model 4 fit statistically significantly (*p* < 0.001) better to the sample than Model 3.

## Discussion

This longitudinal study examined the effects of age, gender, cohort and chronic conditions on the changes in functional mobility among nonagenarians. Our results indicated that the difference in the change in mobility over time for age or gender was not significant. Chronic conditions were significantly related to a change in mobility only among individuals aged 90–91 years.

As there are only a few earlier longitudinal studies on this topic among the oldest-old, it is quite difficult to assess and compare our findings. Our results were in line with the findings of the study carried out by Holstein and colleagues [10]. Findings from this earlier questionnaire-based prospective eight-year follow-up study [10] showed that older age was related to a decline in functional ability in the first four-year follow-up but not in the second four-year period. Based on that observation the authors concluded that age and gender were not related to the eight-year changes in functional ability among 70-95-year-old people. Lamarca and colleagues [15] also observed that functional disability at the baseline was more common among women than among men aged 65 years and older. However, the increase in the rate of functional disability during the eight-year follow-up time, as assessed by the questionnaire, which also included questions about mobility was almost the same: from 42 % to 60 % among women and from 30 % to 48 % among men.

In our study, chronic conditions were significantly related to a change in mobility only among individuals aged 90–91 years, but not in the entire study sample. Health selection could be one potential reason explaining the effect of chronic conditions on mobility changes which was weak when compared to that in younger old people. Individuals with several or serious chronic diseases have a high mortality and they generally have already died at a younger age. Lee and colleagues [16] showed that chronic conditions have lessened the predictive value of mortality among the oldest-old, hence the functional status was a more important predictor of mortality. Our earlier study [17] also showed that functional disability and problems in mobility increased the risk of mortality among nonagenarians. Individuals in our present study were healthier than nonagenarians in general since they had participated in at least two survey rounds and answered the mobility questions. The low number of chronic conditions at the baseline also showed that the participants’ health was quite good. However, there were also individuals who had several diseases. The range of the number of chronic conditions for the participants in the 2001 and 2003 cohorts was from 0 to 5 and for the 2007 cohort from 0 to 6. The number of chronic conditions also varied and increased during the follow-up. A limitation of this study was that we could not show the number of chronic conditions at the follow-up as well. The exact same diseases were not asked about at every time point (2001, 2003, 2007, and 2010) and because of that the chronic conditions at the baseline and follow-up are not exactly comparable.

The range of the chronic conditions for the individuals aged 90–91 years at the baseline was from 0 to 6 (median 1) which is similar to that for the whole sample. However, the effect of chronic conditions on the change in mobility was significant in this sub-group. We did not have data about when the disease was diagnosed or what kinds of problems chronic conditions produced for the activities of daily living. If the medication of the disease is not in balance or a hip fracture has just happened, the effects can be dramatic for a change in mobility and might also have affected our results.

We found statistically significant differences between cohorts in the change in mobility. In the present study the cohort is defined as the year when the participant answered the questionnaire for the first time. The individuals in the 2003 and 2007 cohorts had a significantly faster decline in mobility than individuals in the 2001 cohort. This same cohort effect could also be seen in the sub-analysis among individuals aged 90–91 years (2007 cohort vs. 2001 cohort). More detailed examination between cohorts indicated that the 2001 cohort might not be completely comparable to the 2003 and 2007 cohorts. The age range in the 2001 cohort was wider than the age range in the 2003 and 2007 cohorts, although the median age was the same in all cohorts. However, the differences in the change in mobility could not have been caused entirely by differences in age or the time of the follow-up, because the significant difference between cohorts can also be seen in the individuals aged 90–91 years. Among the individuals aged 90–91 years mobility declined significantly faster among individuals in the 2007 cohort compared with individuals in the 2001 cohort. All in all, the individuals in the 2001 cohort, especially women, more often lived in institutions and more often answered via proxy compared to the individuals in the 2003 and 2007 cohorts. Because the baseline level of mobility was lower in the 2001 cohort, the change in mobility was also minor, and slower when compared with the 2003 and 2007 cohorts. Another explanation for the differences between cohorts in the change of mobility could be the different follow-up time. A majority of the individuals in the 2001 cohort participated for only two years whereas for individuals in the 2003 and 2007 cohorts the follow-up time was longer, lasing three or four years. This difference in follow-up times between individuals in the different cohorts could have exerted an effect on the cohort with respect to the change in mobility.

The advantage of our study is that it is a population-based sample, including both home-dwelling and institutionalized people whose level of mobility varied widely. The data came from one area, the city of Tampere, also included rural areas, and the proportion of inhabitants aged 90 years or older in 2001 was the same as for the country as a whole, 0.46 % [18]. The response rate was high in each wave, and that increases the good generalization of the findings. The same identical questions concerning mobility were used in each survey round to make it possible to faithfully assess the longitudinal changes in mobility.

The number of men in the age group of 90 years and older is commonly low. This small number of men causes difficulties in statistical analysis through limiting the statistical power. However, the percentage of men reflects their share in the basic population, and the participation rate in this study was similar for both men and women. In the present study all information about mobility was based on self-reports. The objectively measured mobility test was able to provide more exact information about the level of mobility. However, mobility tests and measurements carried out for this age group to the extent applied in this case are quite time consuming and financially demanding. The mobility test could thus omit those participants whose mobility is at the lowest level and those who have problems with their mobility and who for that reason can lead to a research bias and restrict the generalization of the results.

## Conclusions

During the follow-up, mobility declined for most of the participants. No differences were observed in the rate of mobility decline over time between age or gender. The effect of chronic conditions on the change in mobility was significant only among individuals aged 90–91 years. The number of the oldest-old people is increasing. Prevention should therefore focus even more on the mobility-related life-style factors that can be modified by oneself, i.e. maintaining muscle strength, in order to maintain independent mobility for as long as possible. Our findings may contribute to our knowledge of the natural history of functional decline among the oldest-old people and influence future work on disability prevention and interventions designed to maintain and improve function.

## Abbreviations

IQR: Interquartile ranges
POR: Proportional odds ratio
ADL: Activities of daily living
SD: Standard deviation
CI: Confidence Interval
BL: Base line
